# Psoriasis, bulbar involvement, and diarrhea in late myoclonic epilepsy with ragged-red fibers-syndrome due to the m.8344A > G tRNA (Lys) mutation

**Published:** 2017-01-05

**Authors:** Josef Finsterer, Gabor Geza Kovacs

**Affiliations:** 1 Krankenanstalt Rudolfstiftung, Vienna, Austria; 2 Institute of Neurology, Medical University of Vienna, Vienna, Austria

**Keywords:** Mitochondrial Disorders, Metabolic Myopathy, Lactate, Epilepsy, Psychiatric, Multi-Organ Disorder Syndrome

Myoclonic epilepsy with ragged-red fibers (MERRF) syndrome was first described by Tsairis, et al. in 1973.^1^ The phenotypic spectrum broadened with the description of > 320 MERRF-patients since then (Table 1).^2^ Here, we report further phenotypic variability. 

The patient is a 71 years, HIV-negative, Caucasian female from nonconsanguineous parents, height 161 cm, weight 55 kg, with a history of a multiple organ disorder syndrome (MODS). Since early childhood she suffered from psoriasis. Since the age of 38 years recurrent diarrhea (1-2 times/day) developed. After a fall over a staircase at the age of 42 years she experienced a traumatic brain injury. Shortly after the trauma she developed left-sided peripheral facial palsy, which never resolved completely. In addition, mild but transient paraparesis of the lower limbs and stocking-type hypesthesia bilaterally were found. Since the age of 53 years bilateral progressive hypoacusis developed. At the age of 54 years diabetes was diagnosed. Since the age of 57 years she noticed myoclonic jerks which could be triggered by light, pain, touch, or fear. Myocloni were associated with recurrent falls (initially 3-4/month, at the age of 58 years 10-15/day) without losing consciousness. Wasting of the entire musculature shortly afterward resulted in weight loss of 10 kg in 1 year. She also reported blurred vision, recurrent double vision, and mild cognitive impairment (MCI) since the age of 57 years. Since the age of 62 years she required nocturnal noninvasive positive-pressure-ventilation and since the age of 65 years mechanical ventilation. Myoclonic seizures resolved only after application of piracetam at the age of 67 years. At the age of 69 years a tracheostoma was implanted. Her last medication included piracetam (24 g/day), glimepiride (1 mg/day), pantoprazole (40 mg/day), fluticasone (3 × 2 H), and bisoprolol (5 mg/day). The patient died at the age of 71 years from gastrointestinal bleeding after implantation of a percutaneous endoscopic gastrostomy. Her history was also positive for anemia, palpitations, hyperlipidemia, hypertension, early morning muscle cramps, easy fatigability, and multiple, large-scale, subcutaneous lipomas in the cervical, shoulder, and thoracic region. Her son had myoclonic epilepsy since the age of 12 years, hypoacusis, and experienced sudden death at the age of 17 years. The father’s family had psoriasis. No information about her mother was available. 

Clinical neurologic exam at the age of 58 years revealed left-sided ptosis, mild wasting of tongue edges, mild dysarthria, left-sided peripheral facial palsy, hypoacusis, weak head anteflexion (M5-), on the upper limbs right predominant diffuse weakness (M5-), diffuse wasting, reduced reflexes, bilateral dysdiadochokinesia, and on the lower limbs proximal weakness, diffuse wasting, absent reflexes, and recurrent myoclonic jerks. She was unable to stand or walk because of sudden loss of muscle tone during myocloni.

**Table 1:** Phenotypic manifestations of the m.8344A > G tRNA (Lys) mutation

**Manifestation**	**Current ** **patient**	**Previously ** **reported** *	**References**
Myopathy	Yes	67	Catteruccia, et al.^3^
Respiratory involvement	Yes	67	Catteruccia, et al.^3^ and Blakely, et al.^4^
Lactate acidosis	Yes	67	Catteruccia, et al.^3^ and Lorenzoni, et al.^5^
Cardiac involvement	Yes	53	Catteruccia, et al.^3^
Polyneuropathy	Yes	47	Catteruccia, et al.^3^
Myocloni	Yes	20-40	Catteruccia, et al.^3^
Epilepsy	Yes	40	Catteruccia, et al.^3^
Cerebellar ataxia	No	13-83	Catteruccia, et al.^3^ and Lorenzoni, et al.^6^
Hypoacusis	Yes	25-35	Mancuso, et al.^2^
Exercise intolerance	Yes	15-25	Mancuso, et al.^2^
Migraine	No	5-15	Mancuso, et al.^2^
Elevated creatine-kinase	Yes	UK	Chinnery, et al.^7^
Elevated CSF protein	No	2-8	DiMauro, et al.^8^
Ptosis	Yes	UK	Blakely, et al.^4^
Cognitive impairment	Yes	UK	Mancuso, et al.^2^
Multiple lipomatosis	Yes	UK	Mancuso, et al.^2^
Diabetes	Yes	UK	Mancuso, et al.^2^
Myalgia	Yes	UK	Mancuso, et al.^2^
Visual impairment	Yes	UK	Chen, et al.^9^
Arterial hypertension	Yes	UK	Austin, et al.^10^
Arrhythmias	Yes	UK	Wahbi, et al.^11^
Optic atrophy	Yes	UK	Mancuso, et al.^2^
Short stature	Yes	UK	Lorenzoni, et al.^6^
Tremor	No	UK	Mancuso, et al.^2^
Leigh syndrome	No	UK	Scalais, et al.^12^ and Monden, et al.^13^
Stroke-like episode/strokeno	No	UK	Vastagh, et al.^14^ and Zaganas, et al.^15^
Leukoencephalopathy	No	UK	Biancheri, et al.^16^
Depression	No	UK	Molnar, et al.^17^
Fibrous bone dysplasia	No	UK	Chen, et al.^9^
Ophthalmoplegia	No	UK	Wiedemann, et al.^18^
Parkinson syndrome	No	UK	Mancuso, et al.^19^ and Horvath, et al.^20^
Pigmentary retinopathy	No	UK	Lorenzoni, et al.^6^
Chronic pancreatitis	No	UK	Toyono, et al.^21^
GI dysfunction**	Yes	UK	Tanji, et al.^22^
Bulbar involvement	Yes	UK	NR
Hyperlipidemia	Yes	UK	NR
Psoriasis	Yes	UK	NR
Diarrhea	Yes	UK	NR

UK: Unknown; NR: Not reported; CSF: Cerebrospinal fluid; GI: Gastrointestinal

* Figures are in percent and relate to the respective size of the cohort investigated,

** Gastrointestinal dysfunction manifested as paralytic ileus.

**Figure 1:**
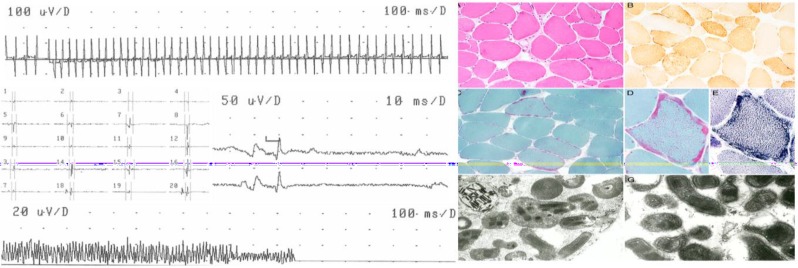
Left part: Myogenic needle electromyography (EMG) of the right anterior tibial muscle showing a pseudomyotonic discharge (upper panel), shortened motor unit action potentials (MUAP) with a mean of 7.9 ms (middle left), satellite potentials (middle right) and a dense, low-amplitude interference pattern (lower panel). Right part: Muscle biopsy from the left deltoid muscle revealed caliber changes and ragged-red fibers in hematoxylin and eosin staining (A), accompanied by cytochrome oxidase-negative fibers (B), Gomori trichrome staining revealed ragged-red fibers (C and D), which were visible in the succinate dehydrogenase enzyme histochemistry staining, (E) electron microscopy confirmed the SS accumulation of mitochondria showed concentrically arrayed tubular cristae and the presence of typical paracrystalline inclusions (F and G)

Blood chemical investigations revealed elevated resting lactate of 2.8 mmol/L (n: < 2.3 mmol/l), hyperlipidemia, fasting blood sugar of 157 mg/dl, and mild hyperCKemia of 113 U/l (n: < 71 U/l). Lactate stress testing was highly abnormal (3.3, 3.6, 6.5, 8.9, 9.2 mmol/l). Nerve-conduction-studies at the age of 58 years revealed reduced nerve conduction velocity (left peroneal nerve). Needle-electromyography (EMG) of the right brachial-biceps-muscle was myogenic with numerous fibrillations and pseudomyotonic discharges, some satellite potentials, mean motor unit action potential (MUAP) duration of 11.4 ms, 15% polyphasia, and a dense, reduced interference-pattern. Needle-EMG of the right anterior-tibial-muscle was myogenic with 4/20 fibrillations, 2/20 pseudomyotonic discharges, a mean MUAP-duration of 7.9 ms, and a dense, low-amplitude interference pattern (Figure 1). Visually-evoked potentials revealed a prolonged P100-latency bilaterally. Cerebral magnetic resonance imaging showed numerous lacunas in the basal ganglia and the left pedunculus cerebri, and mild diffuse atrophy. Electroencephalogram (EEG) at the age of 51 years revealed left predominant generalized spikes and short spike-wave complexes. EEG at the age of 56 years showed recurrently generalized spikes. EEG at the age of 57 years revealed occasional, paroxysmal activity over the frontal projections. Neuropsychological testing demonstrated MCI. Muscle biopsy from the left deltoid muscle at the age of 58 years revealed ragged-red fibers, increase of fat droplets in some fibers, ring-shaped hyperreactive subsarcolemmal (SS) in ragged-red fibers on staining for oxidative enzymes, and some cytochrome oxidase-negative fibers (Figure 1). Electron microscopy showed SS accumulation of mitochondria, increased accumulation of free glycogen, subsarcolemmally and between myofibrils. The mitochondria showed concentrically arrayed tubular cristae and typical paracrystalline inclusions (Figure 1). Genetic testing at the age of 59 years revealed the tRNA (transfer RNA) (Lys) mutation m.8344A > G with a heteroplasmy rate of 70%.

The presented patient is interesting for the multiple organ nature of the phenotype, including the previously unreported phenotypic features psoriasis, chronic diarrhea, bulbar involvement, and hyperlipidemia. Phenotypic manifestations previously reported and present or absent in the presented patient are listed in table 1. Only for some of the previously reported phenotypic manifestations the frequency is known (Table 1).^23^ Her son had developed MERRF-syndrome as well and probably died from sudden cardiac death or sudden unexplained death in epilepsy.

Phenotypic features unreported so far in MERRF-syndrome were hyperlipidemia, psoriasis, bulbar involvement, and diarrhea (Table 1). Whether psoriasis should be regarded as a true manifestation of the disorder remains speculative since she might have inherited it from her father but the m.8344A > G mutation cannot be excluded as cause of the dermatological abnormality. An argument for a causal relation is that dermatological abnormalities are a frequent feature of mitochondrial disorders (MIDs).^24^ Although there are some reports indicating that arterial hypertension could be associated with MID,^25,26^ this association is not generally accepted. Although many patients with MID also manifest with hyperlipidemia, it is not convincing to regard it as a manifestation of the underlying mutation in each case since today hyperlipidemia is endemic in the Western world. However, the high prevalence of hyperlipidemia in MID patients suggests that it can be a phenotypic feature. Bulbar involvement was mild but has not been previously reported in MERRF. An argument for bulbar involvement as a feature of MERRF is that it has been reported in other MID patients.^27^ Although diarrhea has not been reported as a phenotypic feature of MERRF, it is a common feature of other MIDs.^28^ The patient is also noteworthy for the late onset of the syndrome, which usually starts in childhood and rarely in adulthood. The commonly early onset is an argument for psoriasis to belong to the clinical spectrum of MERRF. It is also noteworthy that diarrhea was an early manifestation of the syndrome. The phenotypic variability could be explained with the variable heteroplasmy rates or other modifying factors.

This case shows that the phenotypic spectrum of MERRF syndrome is broader than previously reported and has to be classified as mitochondrial MODS. Diarrhea, psoriasis, bulbar involvement, and hyperlipidemia should be included in the phenotypic spectrum of MERRF-syndrome.

## Notes:

**How to cite this article:** Finsterer J, Kovacs GG. Psoriasis, bulbar involvement, and diarrhea in late myoclonic epilepsy with ragged-red fibers-syndrome due to the m.8344A > G tRNA (Lys) mutation. Iran J Neurol 2017; 16(1): 45-9.
